# PLIP 2021: expanding the scope of the protein–ligand interaction profiler to DNA and RNA

**DOI:** 10.1093/nar/gkab294

**Published:** 2021-05-05

**Authors:** Melissa F Adasme, Katja L Linnemann, Sarah Naomi Bolz, Florian Kaiser, Sebastian Salentin, V Joachim Haupt, Michael Schroeder

**Affiliations:** Biotechnology Center (BIOTEC), CMCB, Technische Universität Dresden, Tatzberg 47-49, 01307 Dresden, Germany; Biotechnology Center (BIOTEC), CMCB, Technische Universität Dresden, Tatzberg 47-49, 01307 Dresden, Germany; Biotechnology Center (BIOTEC), CMCB, Technische Universität Dresden, Tatzberg 47-49, 01307 Dresden, Germany; PharmAI GmbH, 01307 Dresden, Germany; Biotechnology Center (BIOTEC), CMCB, Technische Universität Dresden, Tatzberg 47-49, 01307 Dresden, Germany; PharmAI GmbH, 01307 Dresden, Germany; Biotechnology Center (BIOTEC), CMCB, Technische Universität Dresden, Tatzberg 47-49, 01307 Dresden, Germany

## Abstract

With the growth of protein structure data, the analysis of molecular interactions between ligands and their target molecules is gaining importance. PLIP, the protein–ligand interaction profiler, detects and visualises these interactions and provides data in formats suitable for further processing. PLIP has proven very successful in applications ranging from the characterisation of docking experiments to the assessment of novel ligand–protein complexes. Besides ligand–protein interactions, interactions with DNA and RNA play a vital role in many applications, such as drugs targeting DNA or RNA-binding proteins. To date, over 7% of all 3D structures in the Protein Data Bank include DNA or RNA. Therefore, we extended PLIP to encompass these important molecules. We demonstrate the power of this extension with examples of a cancer drug binding to a DNA target, and an RNA–protein complex central to a neurological disease. PLIP is available online at https://plip-tool.biotec.tu-dresden.de and as open source code. So far, the engine has served over a million queries and the source code has been downloaded several thousand times.

## Graphical Abstract

**Graphical Abstract:**
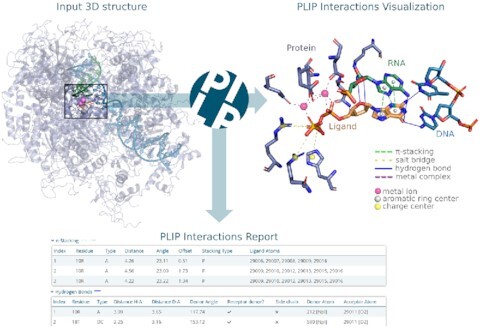
The Protein-Ligand Interaction Profiler. PLIP detects molecular interactions in 3D structures including DNA and RNA.

## MOTIVATION

The Protein Data Bank (PDB) (1) is continuously growing and recent advances in structure prediction and experimental methods such as cryoEM will further accelerate this growth. The vast majority of protein structures in PDB contain ligands and therefore it is important to understand how these ligands interact with their targets.

PLIP, the protein–ligand interaction profiler (2), addresses this need. It detects hydrogen bonds, hydrophobic contacts, π-stacking, π-cation interactions, salt bridges, water bridges, metal complexes and halogen bonds between ligands and targets. PLIP is easy to use as it requires only a PDB ID or a PDB file as input, it has been running reliably and continuously for 5 years, and it is transparent with all the source code published on GitHub. PLIP 2021 constitutes a major update with added support for nucleic acids, more flexibility by adjustable thresholds, mode and model selection and a more functional and modern design for increased usability.

The two main cases for PLIP are the analysis and visualisation of docking results and the in-depth study of existing structures. Elfiky uses PLIP to show docking results of a drug repurposing screen for COVID-19 (3). Furlan *et al.* run an inverse docking screen to identify targets of curcumin, a natural compound (4). They show PLIP interactions for a human folate receptor and a phosphodiesterase. PLIP is used on existing structures by Soliman *et al.* to study antibody recognition of tumour-associated glycans (5) and by Kumar *et al.* to survey specific cation–π interactions (6).

## PLIP WEB SERVER AND COMMAND-LINE

**Figure 1.**
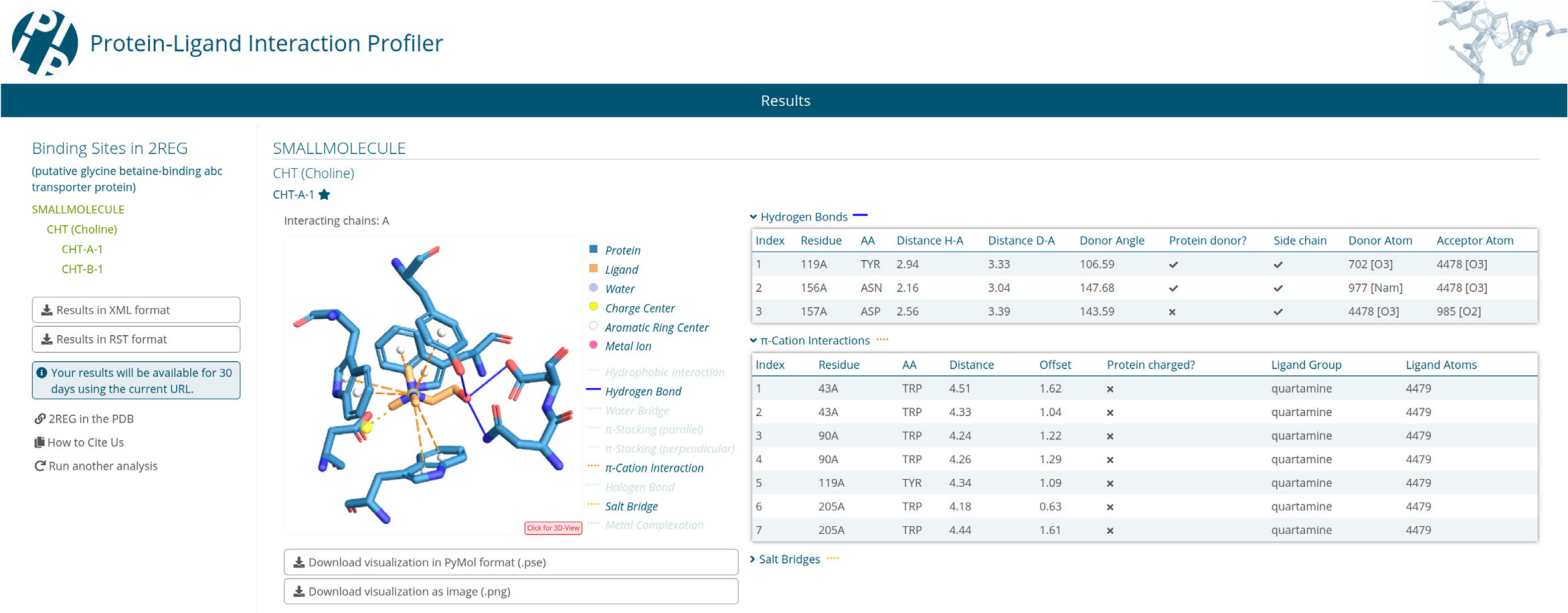
PLIP web tool result page. On the left is a menu for ligands and binding sites, in the middle an image of a selected binding site and on the right a table with details of the interactions.

The PLIP web server provides an easy-to-use interface (Figure 1), which takes as input either a PDB ID or a custom structure in PDB format (e.g. result files from docking or molecular dynamics software). Moreover, the advanced options in the input section allow the users to modify the default settings of PLIP according to their special requirements, such as adjustments to thresholds for the detection of interactions, the consideration of modified residues, the specification of which model to use in multi-model structures, the detection of intra- and inter-chain interactions and the treatment of nucleic acids.

Given an input structure, a list with all binding sites and binding ligands is provided on the left panel of the web tool (see Figure 1). On the right panel, the results for all binding sites are displayed. For each binding site, PLIP offers atom-level binding information, an image, a 3D interactive visualisation with JSmol and a PyMOL session file. In case a manual inspection or subsequent processing is necessary, a parsable XML or RST file with interaction data is available.

The web server is based on the PLIP command-line tools version 2.2.0, which is publicly available at github.com/pharmai/plip under the GNU GPLv2 licence. The command-line tool enables high-throughput computation of protein structures and can be integrated into analysis pipelines using the machine-readable result files. PLIP is easy to use for high-throughput and high-performance computing, as it is virtualised and bundled with all necessary software in Docker and Singularity containers.

## PLIP FOR DNA AND RNA

**Figure 2.**
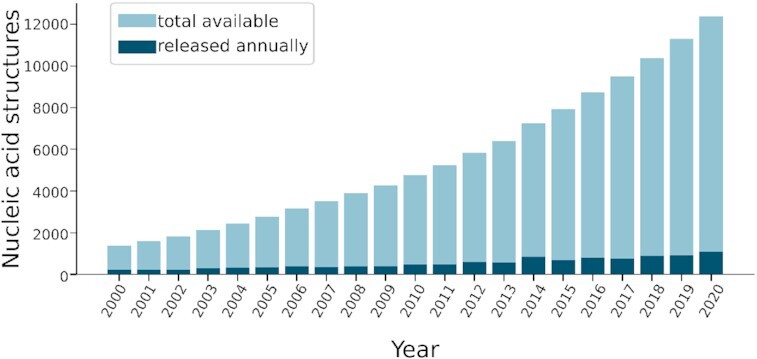
Nucleic acid structures growth in PDB. Number of nucleic acid structures released annually since 2000 and the total aggregated structures.

PLIP’s main focus so far was the detection of interactions between small molecules and proteins. However, with the constant growth of nucleic acid structure data, a large proportion of over 7% of the PDB (12460 structures) contains DNA and RNA (Figure 2). Of these, the majority are protein–nucleic acid complexes (8837) and the rest are mostly DNA or RNA only.

Studying the interactions of nucleic acids to ligands and to proteins is important. DNA/RNA can act, for instance, as a target for novel cancer drugs such as the drug XR5944 (7) and as a ligand to proteins involved in disease such as the protein FUS, which is implicated in the neurological disorder ALS (8).

### Protein–RNA interaction: FUS binding UGGUG

**Figure 3.**
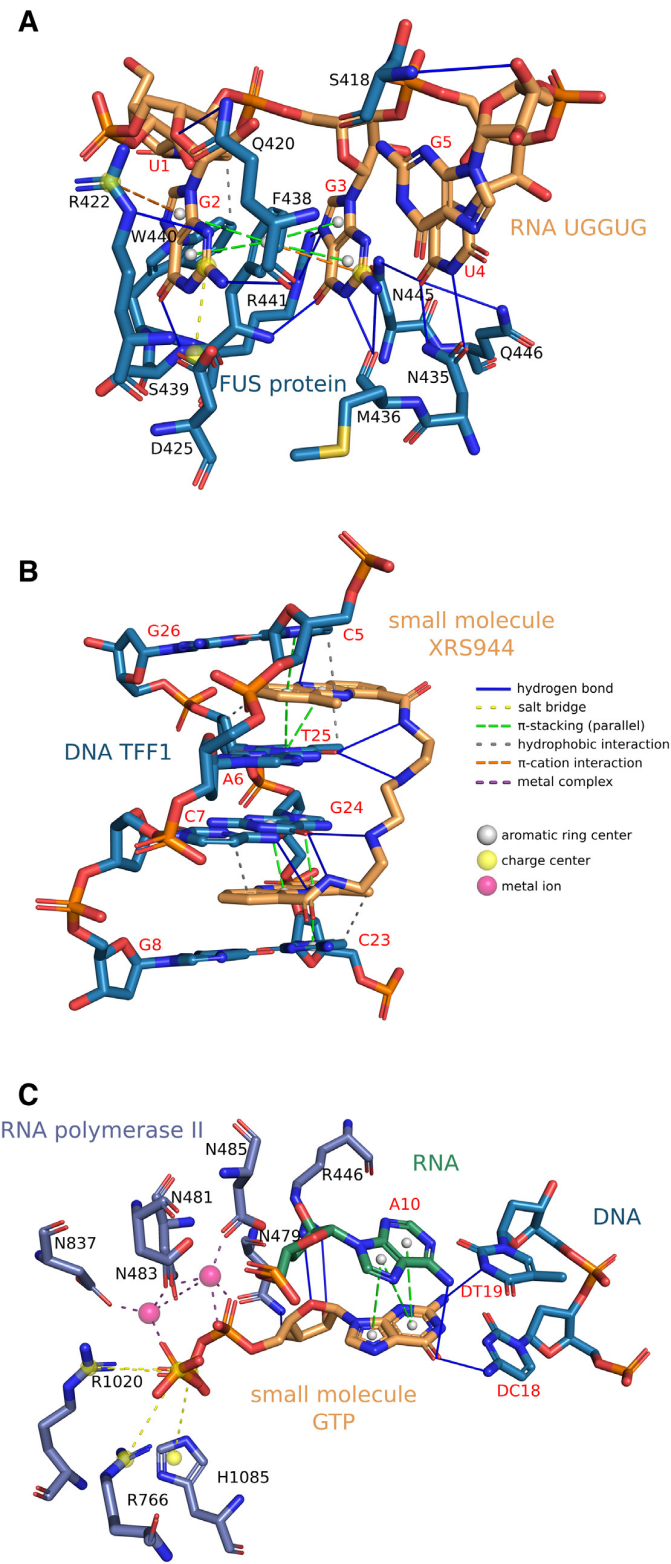
Examples. (**A**) FUS binding the UGGUG (PDB ID: 6G99), (**B**) XR5944 binding the TFF1 estrogen response element (PDB ID: 2MG8) and (**C**) GTP binds RNA polymerase II and DNA/RNA of the elongation complex (PDB ID: 2E2H). Ligands are shown in orange and receptors in blue, green or purple gray. Protein residues are labeled in black and DNA/RNA bases in red.

FUS is a nuclear RNA-binding protein involved in RNA metabolism. It can undergo liquid–liquid phase separation, forming membraneless organelles (9–11). Aberrant phase transition of FUS into pathological solid aggregates has been linked to neurodegenerative diseases (12,13). Recent studies have shown that high RNA concentrations prevent phase separation of FUS (8) and mutations in FUS can cause aberrant phase separation (14). Loughlin *et al.* have solved the structure of FUS bound to RNA (15). We applied the new version of PLIP to identify the interactions between the zinc finger of FUS as receptor and the RNA sequence UGGUG as ligand (Figure 3A). PLIP detects π-stacking interactions of the central Phe438 with the flanking G2 and G3 of the RNA. G2 is additionally anchored by a π-cation contact with Arg422, a salt bridge with Asp425, and several hydrogen bonds with Arg422, Trp440, Ser439 and Gln420. Multiple sequence-specific hydrogen bonds are also formed between G3 and Phe438, Arg441 and Met436. U4 binds the FUS zinc finger via hydrogen bonds with Asn435, Gln446 and Asn445. The zinc finger recognises U1 by a hydrophobic interaction. Illuminating the RNA-binding mode of FUS paves the way for a deeper understanding of the mechanisms that lead to the formation of solid aggregates in neurodegenerative diseases.

### Small molecule–DNA interaction: XR5944 binding estrogen response element

The cancer drug candidate XR5944 is a DNA bis-intercalator that strongly binds DNA in a sequence-specific manner (7,16–18). Because of its structural similarity to other topoisomerase inhibitors, XR5944 was originally thought to inhibit topoisomerase I and II (19), but was later shown to be a DNA transcription inhibitor instead (20,21). Interestingly, XR5944 specifically recognises the estrogen response element (ERE), the target DNA sequence of estrogen receptor-α (ERα), making it a promising drug for the treatment of ERα-positive breast cancer (18,22,23). EREs are present in the regulatory region of several genes, including Trefoil factor 1 (TFF1). Lin *et al.* published the structure of two XR5944 molecules bound to the naturally occurring DNA TFF1-ERE (18). Thus, TFF1 acts as receptor and XR5944 as ligand, which PLIP treats accordingly (Figure 3B). PLIP identifies parallel π-stacking interactions between the phenazine rings of the XR5944 molecules and the flanking base pairs. In addition, it detects several hydrophobic interactions at the intercalation sites. Furthermore, the carboxamide aminoalkyl linkers of the XR5944 molecules form site-specific hydrogen bonds with the base pairs in the major groove. The non-covalent interactions formed by XR5944 with the TFF1-ERE determine its binding mode and sequence specificity. Uncovering these interactions provides a structural basis for the rational design of drugs that target EREs.

### Small molecule–protein/DNA/RNA interaction: GTP binding RNA polymerase II and the DNA/RNA strands

Transcription, the synthesis of RNA from a DNA template, is one of the most important steps in the control of cell growth and differentiation (24,25). During transcription, the information in a strand of DNA is copied into a new molecule of mRNA, mainly carried out by the enzyme RNA polymerase II and a number of accessory proteins called transcription factors (26). The RNA polymerase begins mRNA synthesis by unwinding the DNA helix and adding complementary bases to the RNA strand (elongation). The substrates for RNA synthesis are the four nucleoside triphosphates ATP, GTP, CTP and UTP. Cleavage of the high-energy phosphate bond between the phosphate groups in the nucleoside triphosphate structure provides the necessary energy for the addition of nucleotides into the growing RNA chain (27). Wang *et al.* obtained the X-ray structure revealing the transcribing complex in the ‘post-translocation’ state with the nucleotide added to the RNA transcript (28). Figure 3C shows how PLIP analyses such a complex and characterises the binding of GTP (orange) to the RNA polymerase (purple grey), RNA strand (green) and DNA strand (blue), all at the same time. The guanine group in GTP binds to the DNA strand with three hydrogen bonds and to the RNA strand via parallel π-stacking and one hydrogen bond. Moreover, the phosphate groups in GTP bind to the RNA polymerase residues via a salt bridge and a metal complex. The characterisation of the GTP binding mode in the elongation complex provides a structural understanding of the transcription mechanism.

### PLIP algorithm for DNA/RNA detection

PLIP uses four steps to detect and report the relevant interactions of a complex: structure preparation, functional characterisation and rule-based matching and filtering of interactions. In the preparation step, the input structure is hydrogenated and the ligands are extracted along with their binding sites. If the advanced option for analysing DNA or RNA as receptor is selected, PLIP will automatically detect the nucleic acid residues as part of the receptor and exclude them from the ligand molecules. The RNA and DNA residues to be considered are detected by their name U, A, C, G and DT, DA, DC, DG, respectively. After the correct detection of receptor and ligands, PLIP continues with the subsequent interaction detection and visualisation as previously described in (2).

## PLIP AND OTHER TOOLS

There are a number of tools (29–37) analysing ligand–target interactions, but apart from one, they have a different focus and therefore suffer from some limitations: (29–33) detect only a limited set of interaction types and do not offer 3D visualisation, (34) requires extensive preparation of input files, (38) is only commercially available and (35,36) are limited to binary interaction fingerprints and do not cover DNA and RNA. Arpeggio (37) is most closely related to PLIP offering comparable functionality except for flexible adjustment of thresholds.

## CONCLUSION

Interactions between nucleic acids and small molecules play a crucial role in various biological processes and have a strong impact on drug discovery. In that regard, the PLIP web tool has been adapted to recognise nucleic acid molecules as receptors and provide a comprehensive analysis and visualisation of non-covalent interactions with a one-click loading of structures. Moreover, its novel design displays a user-friendly platform, which facilitates the intuitive use of the tool’s features and exploration of the results for a better understanding of the biological processes behind the binding mechanism. Furthermore, the availability of PLIP source code enables local batch processing, customisation of the algorithm for special applications as well as active development of the tool in the community.
